# Why Are There Failures of Systematicity? The Empirical Costs and Benefits of Inducing Universal Constructions

**DOI:** 10.3389/fpsyg.2016.01310

**Published:** 2016-08-31

**Authors:** Steven Phillips, Yuji Takeda, Fumie Sugimoto

**Affiliations:** ^1^Mathematical Neuroinformatics Group, Human Informatics Research Institute, National Institute of Advanced Industrial Science and TechnologyTsukuba, Japan; ^2^Automotive Human Factors Research Center, National Institute of Advanced Industrial Science and TechnologyTsukuba, Japan

**Keywords:** systematicity, compositionality, universal construction, association, classicism, connectionism, learning

## Abstract

Systematicity is a property of cognition where capacity for certain cognitive abilities implies capacity for certain other (structurally related) cognitive abilities. This property is thought to derive from a capacity to represent/process common structural relations between constituents of cognizable entities, however, systematicity may not always materialize in such admissible contexts. A theoretical challenge is to explain why systematicity fails to materialize in contexts that allow the realization (e.g., by induction) of common structure (universal construction). We hypothesize that one cause of failure arises when the potential gain afforded by induction of common structure is overshadowed by the immediate benefit of learning the task as independent stimulus-response associations. This hypothesis was tested in an experiment that required learning two series of pair maps that involved products (universal construction), or non-products (control) of varied size: the number of unique cue/target elements (three to six) constituting pairs. Each series was learned in either ascending or descending order of size. Only performance on the product series was affected by order: systematicity was obtained universally in the descend group, but only on large sets in the ascend group, as revealed by the significant order × size interaction for errors in the product condition, *F*_(3, 87)_ = 3.38, *p* < 0.05. Smaller maps are more easily learned without inducing the common product structure, which is more readily observable with larger maps: larger maps provide more evidence for relationships between stimulus dimensions that facilitate the discovery of the common structure. The new challenge, then, is to explain the systematic learnability of stimulus-response maps, i.e., second-order systematicity.

## 1. Introduction

An important feature of cognition is that possession of cognitive capacities appears to be organized on the basis of common “structures.” That means, for example, if a person has the ability to recognize the square as being the left object in a scene consisting of a square to the left of a triangle, then that person has the ability to recognize the triangle as being the left object in a scene consisting of a triangle to the left of a square. Put another way, in general, people don't have one of those two capacities without having the other. Both capacities are related by a common structure: the spatial relation relationship between the two objects. This property of cognition is called *systematicity* (Fodor and Pylyshyn, 1988). Systematicity has been more formally characterized as having cognitive capacity *c*_1_ if and only if having structurally related cognitive capacity *c*_2_, i.e., as structural equivalence classes of cognitive capacities (McLaughlin, 2009).

The systematicity challenge for cognitive science is to explain *why* cognitive capacity is distributed along common structural relations. That is to provide a theory of cognitive architecture—the basic cognitive representations and processes, and their modes of composition—from which systematicity properties *necessarily* follow (Fodor and Pylyshyn, 1988; Aizawa, 2003). The classical (symbol systems) theory of cognitive architecture supposes *symbolic* cognitive processes for constructing and operating on symbolic representations of the world. For example, the scene consisting of a square to the left of a triangle is represented by a pair of symbols, *st*, where the first symbol, *s*, represents the square and the second symbol, *t*, represents the triangle so that the spatial relationship between square and triangle is *mirrored* by the syntactic relationship between the corresponding symbols representing those constituent entities. Assuming a symbolic process, *first*, for accessing the first symbol of a pair of symbols, then systematicity follows from the fact that the inferences pertaining to the square-triangle and triangle-square scenes involve one and the same process, i.e., *first*(*st*) = *s* and *first*(*ts*) = *t*; there is no case of having one inferential capacity without having the other. An analogous arrangement of neural processes can be developed for a connectionist theory to likewise demonstrate systematicity (Smolensky, 1987).

The problem for both approaches, however, is the lack of clear theoretical criteria from which systematicity is a necessary consequence (Aizawa, 2003). The connectionist approach of positing networks of weighted connections between neurons was criticized, as a *theory* of cognitive architecture, because this kind of theory admits models with and without the requisite systematicity properties (Fodor and Pylyshyn, 1988; Fodor and McLaughlin, 1990). Systematicity does not necessarily follow from such connectionist theory. Ironically, the same problem befalls the classical approach, as one can configure a symbol system with grammatical rules that do or do not support systematicity (Aizawa, 2003; Phillips and Wilson, 2010). To obtain systematicity, classicists assume only “canonical” grammars, i.e., the grammars that support systematicity (McLaughlin, 2009). However, this assumption appears to be *ad hoc*: motivated only to fit the data, not confirmable independently of confirming the theory, and unconnected to the rest of the theory's core principles (Aizawa, 2003). So, it remains unclear how classical theory is supposed to fully explain systematicity (Aizawa, 2014).

To overcome these shortcomings, we proposed a *category theory* (Mac Lane, 1998) approach to cognitive architecture, whereby a systematicity property is explained as a necessary consequence of a categorical universal construction (Phillips and Wilson, 2010, 2011, 2012). Our category theory approach starts with the formal, mathematical concept of a category, which consists of a collection of *objects*, a collection of relations between objects, called *morphisms*, and a composition operation that takes two morphisms and returns a morphism. A common first example is **Set**, the category of sets (objects) and functions (morphisms). Here, we interpret morphisms as cognitive processes (functions) between sets of cognitive representations, and the composition operation is composition of cognitive processes. A universal construction is an arrangement whereby each morphism in a particular collection of morphisms is obtained by the composition of a common (*universal*) morphism and a unique morphism. For intuition, in regard to the square-triangle example, one can think of the universal morphism as the common relational schema and each unique morphism as the unique combination of constituents in that relation. Composition of each unique morphism with the universal morphism yields no fewer (necessity) and no more (sufficiency) than one morphism for each relational instance. Thus, systematicity is a necessary and sufficient consequence of universal construction (see Phillips and Wilson, 2014, for a summary).

The intuitive example just given alludes to an important universal construction, called the categorical *product*, that we use throughout this paper. In general, a categorical product of two objects, *A* and *B*, consists of the product object, *P*, and two morphisms, *p*_1_:*P* → *A* and *p*_2_:*P* → *B*, that recover the *A* and *B* constituents. In **Set**, the categorical product is the *Cartesian product* of two sets *A* and *B*, which is the set of all pairwise combinations of elements from each set, i.e., the set {(*a, b*)|*a* ∈ *A, b* ∈ *B*}, and two functions, called projections, that return the first and second components of each pair, i.e., π_1_(*a, b*) = *a* and π_2_(*a, b*) = *b*. We have argued that categorical products underlie a variety of relational (Phillips and Wilson, 2010) and inferential cognitive capacities (Phillips et al., 2009), and shown that changes in product arity, supposed to underlie visual search difficulty, correspond to changes in EEG synchrony (Phillips et al., 2012). In each case, the response depends on representing combinations of components in a way that maintains the identity of each component, which is afforded by the projections. In conjunctive visual search, for example, the target of search is uniquely identified by a combination of item features, such as color and orientation (binary product), or color, orientation, and spatial frequency (ternary product). These results suggest that products are fundamental to many cognitive tasks, where constituents must be combined in ways that maintain their identities.

### 1.1. Absence of systematicity

Although systematicity is a property of some aspects of cognition, systematicity may not materialize in other situations. These situations also attest to common structure as the basis for equivalence classes of cognitive capacities. Idioms, such as “John kicked the bucket”—John died—are examples (Fodor and Pylyshyn, 1988, p. 42). Whereas, the systematicity of “John kicked the ball” and “John kicked the bat” accords with the common structural relation, *kicked*, the lack of systematicity with regard to the idiom “John kicked the bucket” accords with the fact that understanding this idiom depends on understanding a different relation, i.e., *died*. Hence, while you don't find English speaking people who understand the meaning of “John kicked the ball” but not “John kicked the bat,” you do find English speakers who understand the meaning of “John kicked the ball” but not the idiomatic meaning of “John kicked the bucket,” even though they understand the meanings of constituents, “John,” “bat,” “ball,” “bucket,” and “kicked.” Absence of systematicity is further exemplified by the “phrase book” model of language (Fodor and Pylyshyn, 1988, pp. 37–38), where say a tourist grapples with communication in a foreign language by committing to memory a collection of common-use phrases. Whether or not phrases are committed to memory is independent of their structural relations, which accords with the tourist's lack of systematic comprehension of that language.

The category theory explanation says that universal constructions are necessary and sufficient for systematicity. Therefore, by this explanation, absence of systematicity is implied by failure to possess the requisite universal construction. For idioms, absence of systematicity is straightforwardly due to the fact that there are no common structural relations to be represented/processed, which in categorical terms means that there does not exist a universal morphism. In the phrase book example, however, the common structural relation is present, but is not represented by the tourist's rote memorization approach to language acquisition: each phrase is committed to memory irrespective of its grammatical structure. This situation raises a more general question for theories of cognitive architecture, including the categorical theory: Why do people fail to represent/process an existing common structural relation? In categorical terms, this question is to ask why the existing universal morphism is not represented.

Part of the reason for the absence of systematicity in the phrase book example is that the phrases acquired by rote learning are superficially represented as sequences of otherwise unstructured letters/words that have no further meaning to the tourist. Naturally, a theory of cognitive architecture can account for these exceptions by simply representing them as such. The deeper and more general question, however, asks why the cognitive system represents such structurally related entities without choosing to represent their common structure. The intuitive reason behind the phrase book example, which motivates the current study, is a kind of cost-benefit tradeoff: a tourist may decide that the benefits of proficiently using a foreign language for a few days are outweighed by the cost (months/years) required to learn that language to an adequate level of proficiency. In this case, one may simply learn the surface features, without grasping the deeper grammatical structure.

Universal constructions are amenable to a cost-benefit analysis, because every morphism pertaining to the universal construction is equally a composition of two morphisms: the universal morphism and the unique morphism. A simple analysis of Cartesian products, i.e., products in **Set**, suggested a cost-benefit tradeoff in regard to morphisms modeling maps between sets of cue/target stimuli (Phillips, 2013). A more detailed introduction is provided in the next section, which motivates the design of the current experiment. The basic intuition given here is that when the number of mapped elements is small, it may be more cost-effective to learn mappings as direct links from cues to targets, rather than indirectly via links to/from the underlying product. Conversely, when the number of mappings is large, it may be more efficient to learn each mapping as a composition of mappings that involve the product. This intuition parallels that for the phrase book example: when the number of occasions that require use of the foreign language are likely to be few, rote learning may be more cost-effective. However, a rote learning approach is prohibitively expensive for everyday use.

The purpose of the current study is to test the behavioral implications of learning universal (product) constructions, and thereby provide empirical support for our universal constructions explanation for systematicity and the cost-benefit hypothesis. To facilitate exposition, we introduce just the basic constructions (functions) needed for the experimental design. The basic category theory upon which the hypothesis is based is given in Phillips (2013). For further theoretical details on the category theory (universal constructions) approach to systematicity see Phillips and Wilson (2010), or Phillips and Wilson (2014) for an overview. Introductions to category theory can be found in many books on this topic (e.g., Mac Lane, 1998; Awodey, 2010; Simmons, 2011; Leinster, 2014). For applications of category theory to other areas of cognitive science (see e.g., Halford and Wilson, 1980; Magnan and Reyes, 1995; Ehresmann and Vanbremeersch, 1999; Lambek, 2004; Ellerman, 2007; Healy et al., 2008; Gómez-Ramirez and Sanz, 2011; Phillips, 2014). General implications of this categorical perspective on acquisition of systematicity are also discussed in the final section.

### 1.2. Categorical product: behavioral implications

In this section, we introduce a concrete example of a categorical product to test the behavioral implications of our category theory approach to systematicity and failure. The example involves products of sets and associated functions in the context of learning maps from stimulus cues to targets.

A collection of cue-target mappings can be modeled as a function from a set of cues to a set of targets. For example, suppose the cues are characters G, K, and P, and corresponding targets are shapes ★, ▼, and ♣, respectively. This collection of cue-target mappings is modeled as the function *char2shape*:*Char* → *Shape*; *G* ↦ ★, *K* ↦ ▼, *P* ↦ ♣, where *Char* is the set of character cues, and *Shape* is the set of shape targets, and the “maps to” symbol (↦) indicates a specific cue-target mapping.

Targets may be compositional. Suppose the targets also have a color feature, respectively, red, green, and blue. In this case, each character also cues a target color, modeled as the function *char2color*: *Char* → *Color*; *G* ↦ red, *K* ↦ green, P ↦ blue, where *Color* is the set of color features. So, each character cues a pair of features, modeled as the function 〈*char2color*, *char2shape*〉:*Char* → *Color* × *Shape*; *G* ↦ (red, ★), *K* ↦ (green, ▼), P ↦ (blue, ♣).

**Figure 1:**
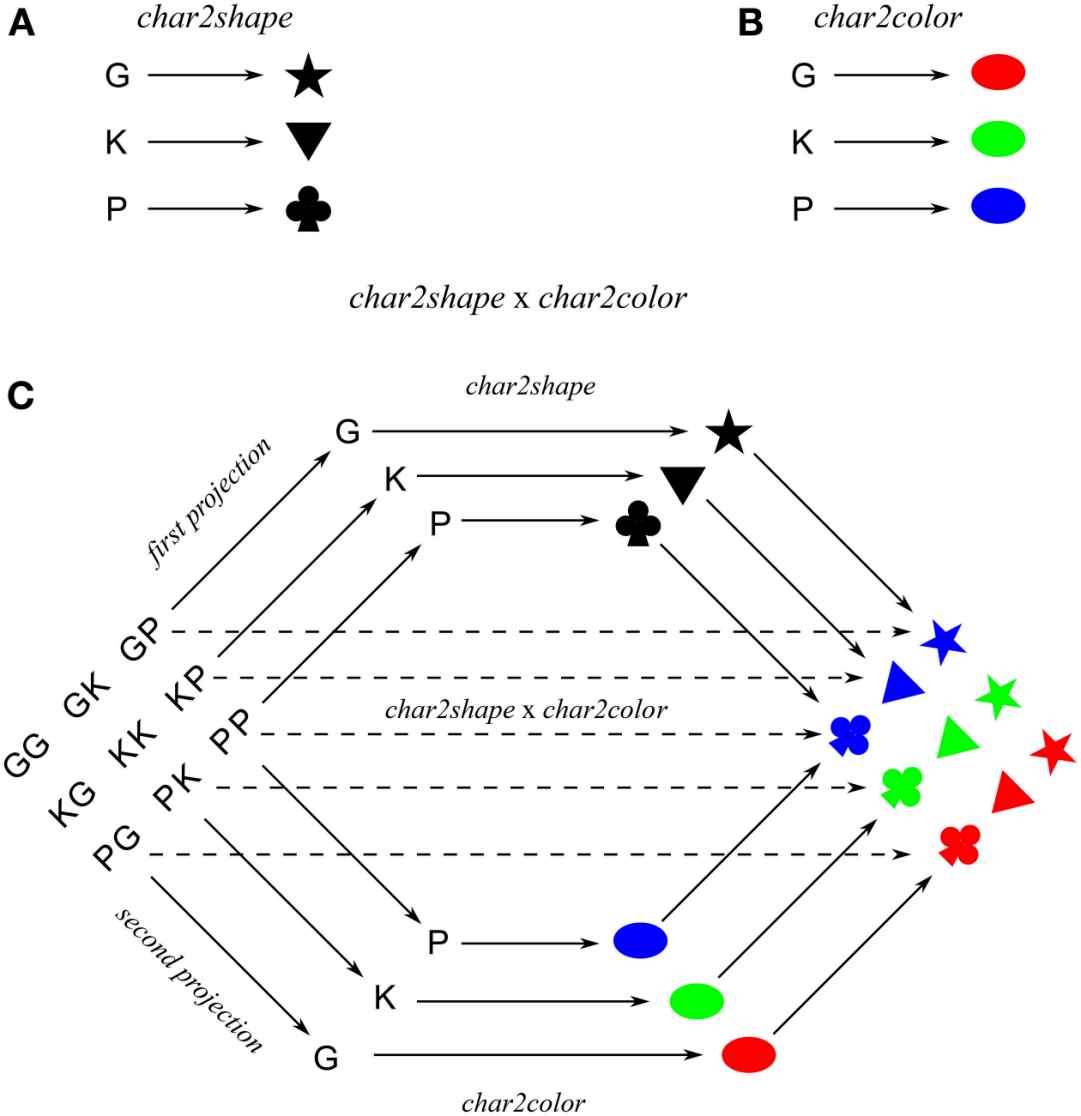
**Maps (A) *char2shape* and (B) *char2color* and their product (C) *char2shape* x *char2color***. The dashed arrows direct computation of the product map, solid arrows indicate indirect computation of the product map via the two component maps.

Cues and functions may also be compositional. For example, the *product* of functions *char2color* and *char2shape* is the function *char2color* × *char2shape*: *Char* × *Char* → *Color* × *Shape*, which sends each pair of characters to a (color, shape) pair in accordance with the constituent functions, e.g., (*G, G*) ↦ (red, ★), (*G, K*) ↦ (red, ▼), etc. The nine *char2color* × *char2shape* mappings are obtained from the product of the three *char2color* mappings and the three *char2shape* mappings. Figure 1 shows *char2color* and *char2shape*, which consist of three mappings each, and their product *char2color* × *char2shape*, which consists of nine mappings composed from the six component mappings, i.e., the three mappings from *char2color* and the three mappings from *char2shape*. In general, the product of maps *f*:*A* → *C* and *g*:*B* → *D* each consisting of *n* mappings (i.e., 2*n* mappings in total) is the map *f* × *g*:*A* × *B* → *C* × *D*; (*a, b*) ↦ (*f*(*a*), *g*(*b*)) which consists of *n*^2^ mappings. Hence, computing a mapping via a product (if it exists) offers greater benefit with larger *n*.

Induction of a product of functions is also possible from a subset of its mappings. For example, suppose one is given the *char2color* × *char2shape* mappings (*K, G*) ↦ (green, ★), (P, G) ↦ (blue, ★), (G, K) ↦ (red, ▼), and (G, P) ↦ (red, ♣), where each constituent *char2color* and *char2shape* mapping appears at least once in the set. Having induced the constituent maps, one can then infer the other *char2color* × *char2shape* mappings from the product of those constituent maps. We refer to this form of induction as *behavioral generalization*. For comparison, in the context of language, this form of generalization is called *weak systematicity* (Hadley, 1994).

Behavioral generalization is not afforded by every function from *A*×*B* to *C* × *D*. In particular, the function 〈*p, q*〉:*A* × *B* → *C* × *D*, where *p*:*A* × *B* → *C* and *q*:*A* × *B* → *D* does not afford generalization when each target component is uniquely determined by each pair of cues (Note that a map *f* × *g* is called a *product of functions*, and a map 〈*p, q*〉 is called a *product function*. We refer to the latter as a *non-product function/map*, meaning *not* a product of functions, to avoid confusion).

As already mentioned, previous work (Phillips, 2013) suggested that construction of products depends on a cost-benefit tradeoff: when the number of mappings is small the cost of constructing products may outweigh the benefit (response prediction); conversely, when the number of mappings is large benefit may outweigh cost. In terms of behavioral generalization, then, no behavioral generalization is predicted for product maps consisting of a small number of mappings (capacity for the associated universal morphism is absent), but behavioral generalization is predicted for product maps consisting of a large number of mappings (capacity for the associated universal morphism is present). The purpose of the current study in to test these predictions.

The hypothesis is that systematicity fails to materialize when the cost of (learning to) represent a universal construction outweighs its (perceived) benefits, such as correct responses to subsequently related task stimuli. One difficulty with testing a potential tradeoff over universal constructions is that we do not have an independent measure of the cost of inducing/representing a product. To circumvent this difficulty, we test participants on products of different sized maps. Accordingly, we expect that as the size of the constituent maps increases, participants will exhibit a transition from computing the product map directly, i.e., without making use of the underlying universal construction (product), in which case there will be no behavioral generalization, to computing the map indirectly via the product construction, in which case there will be behavioral generalization. Figure 1C indicates the direct route as dashed arrows and the indirect route as solid arrows. Conversely, for participants initially given products of large maps, we expect that they will use the product construction. Since these participants have already induced the product structure, which can then be employed for products of smaller maps, we expect behavioral generalization for products of all sized maps in this case. So, in the experiment that follows, one group of participants is tested on a series of product maps in the order of small to large (*ascend* group), and the other group of participants is tested on the same series of product maps, but in the order of large to small (*descend* group). The prediction is that the ascend group will exhibit increased behavioral generalization on product maps with increased map size, but the descend group will exhibit behavioral generalization on product maps across all sizes.

## 2. Methods

Participants were required to learn two cue-target maps: (1) a *product* map, *f* × *g*:*A* × *C* → *B* × *D*, which consists of maps *f* : *A* → *B* and *g* : *C* → *D*, as the experiment condition; and (2) a *non-product* map, 〈*p, q*〉 : *A* × *C* → *B* × *D*, that had no such product composition, as a control condition. In each case, participants were first trained on a subset of mappings, followed by testing on all mappings. Our primary interest is whether they learned to decompose the product map *f* × *g* into its constituents *f* and *g*.

### 2.1. Participants

There were 31 adults who were paid a flat rate of 5000-yen, regardless of performance, to participate in the experiment: 19–40 years of age (23.2 mean), 7 female. All were right-handed with normal or corrected-to-normal vision. This study was carried out in accordance with the recommendations of “Guidelines for handling ergonomic experiments, Committee on Ergonomic Experiments, Bioethics and Biosafety Management Office, Safety Management Division, National Institute of Advanced Industrial Science and Technology” with written informed consent from all participants. All participants gave written informed consent in accordance with the Declaration of Helsinki.

### 2.2. Apparatus and stimuli

**Figure 2:**
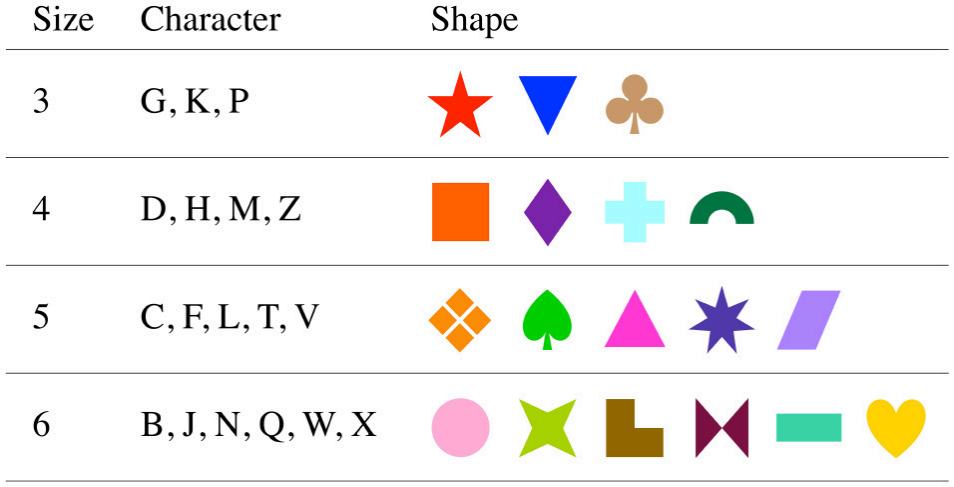
**Cue and target stimuli**.

Stimuli were presented by a notebook computer (Mac OSX) using MATLAB software (MathWorks, Natick, MA) with the Psychophysics Toolbox extensions (Brainard, 1997; Kleiner et al., 2007) on an external display, 43 cm (width) and 33 cm (height), placed about 57 cm from the participant, so 1 cm is ~1° field of view. The shape stimuli were chosen from shape materials in Microsoft Office (Microsoft, Redmond, WA), and their colors were defined by web safe color codes. The approximate angle (width × height) subtended of each string and shape were 5° × 1.5° and 2° × 2.4°, respectively. Screen resolution was 1920 × 1200 pixels; refresh rate was 60 Hz. Stimuli were displayed on a gray background. Stimuli are shown in Figure 2.

### 2.3. Conditions

We used a factorial design with the following factors.

*Order*: Participants where divided into two groups. The *ascend* group (15 participants, 3 females) did each task in the order of smallest to largest number of task mappings; the *descend* group (16 participants, 4 females) did each task in reverse order.
*Task*: Task structure was a *product*, or a *non-product*.
*Size*: The number of unique character to color/shape mappings, *n*, was three, four, five, and six.
*Cue*: For the testing stage, a pair cue was either *old*, if the characters appeared together as a pair cue in the training stage, or otherwise *novel*. All pairs presented in the training stage were considered old.

### 2.4. Procedure and analysis

For each participant we have the following procedure.

**Figure 3:**
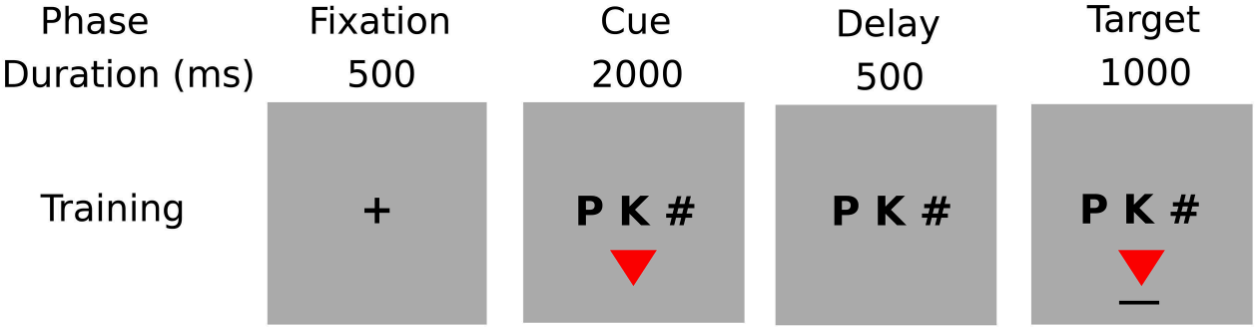
**Phases of a training trial**.

*Experiment*: Each experiment consisted of eight sessions, one session per set size for the product task, and one session per set size for the non-product task. Sessions are grouped by task. Task and size order were randomized.
*Session*: Each session consisted of sequence of training blocks, followed by one test block. Training ceased when the minimum 90% correct response (one block) criterion was reached, after administering five blocks.
*Training/testing block*: Each training block consisted of $\frac{1}{2}n^{2}$ training trials (i.e., 4, 8, 12, 18 trials), balanced over features; each testing block consisted of *n*^2^ trials, i.e., all cue-target pairs.
*Training/testing trial*: Each training trial consisted of four phases: fixation (500 ms), cue/shape presentation/response (2000 ms), delay (500 ms), target feedback (1000 ms); each testing trial consisted of two phases: fixation (500 ms), and cue/shape presentation/response (2000 ms), i.e., no feedback (see Figure 3).
*Cue/shape and target feedback displays*: The Cue/shape display consisted of a string of three characters: two characters constituted the pair cue, and one hash character (“#”) was to be ignored, and a colored shape. During presentation participants were required to respond by pressing the “Yes” key when the displayed shape was the target for the given cue, otherwise by pressing the “No” key. Participants were instructed to respond as accurately and quickly as possible. The target/feedback display consisted of the same three characters as cue/shape display, and the target colored shape associated with the given cue, underlined to indicate feedback. Training and testing trials were self-paced: each trial was initiated by pressing a key.

The order of task instances was randomized. Within-block trial order was also randomized. Participant response errors and times were recorded. Analyses of variance (ANOVAs) were conducted on error rates to access effect significance; *t*-tests were conducted on error rates to determine whether performance was significantly about chance level (50%). Upon completion of each series of tasks, we asked participants to report on the method they used to map cues to targets.

## 3. Results

Our primary interest is performance on (novel) test trials, as a measure of behavioral generalization. So, after reporting analysis for all training and testing trials, we report test trial analyses for novel and old trials, and product and non-product conditions. We also report on participant awareness of the structure underlying the product task.

### 3.1. Training

#### 3.1.1. Training blocks

**Figure 4:**
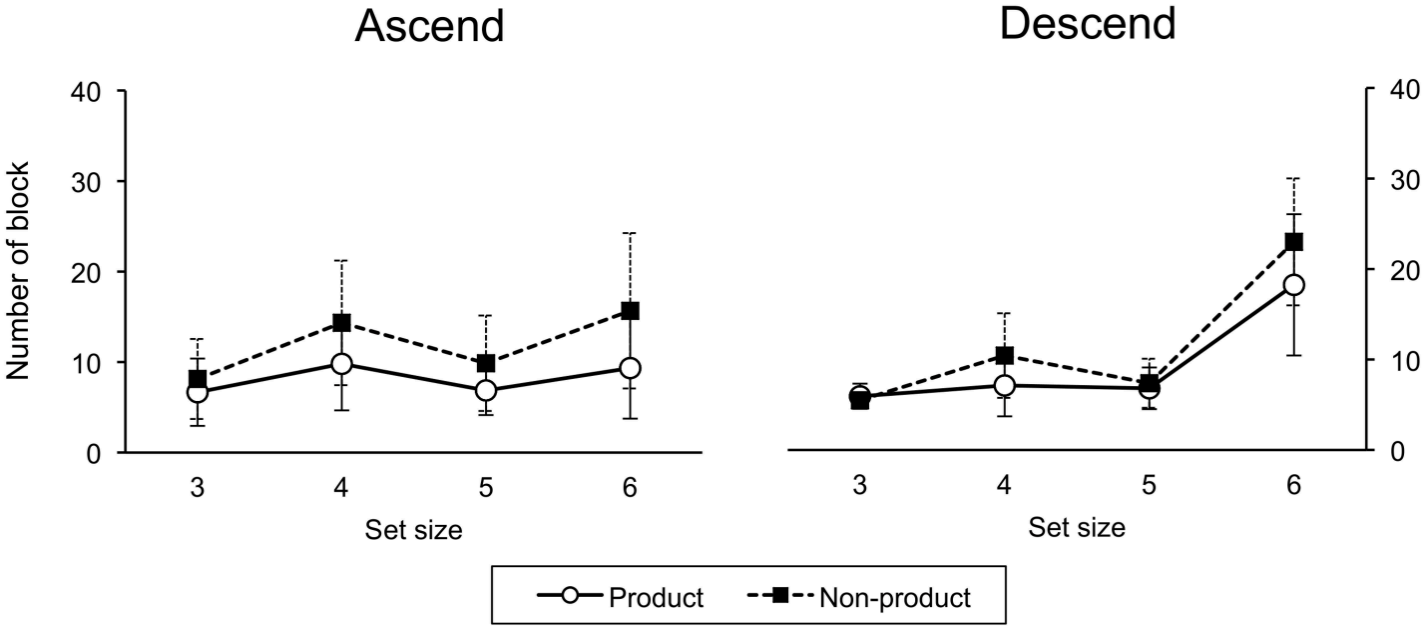
**Number of training blocks to criterion**. Error bars indicate one standard deviation.

A three-factor (order, task, size) ANOVA on number of blocks to criterion revealed a main effect of task, *F*_(1, 29)_ = 21.75, *p* < 0.001. More training blocks were required to reach criterion for the non-product than product task. There was a main effect of size, *F*_(3, 87)_ = 65.73, *p* < 0.001. Fewer blocks were required at size 3 than sizes 4, 5, and 6; and for size 5 than sizes 4 and 6. There was a significant order-size interaction, *F*_(3, 87)_ = 22.08, *p* < 0.001 (Figure 4).

#### 3.1.2. Ascend and descend order

For the ascend group, a two-factor (task, size) ANOVA revealed main effects of task, *F*_(1, 14)_ = 18.10, *p* < 0.001, with more blocks in the non-product condition, and size, *F*_(3, 42)_ = 10.04, *p* < 0.001. Likewise, for the descend group, there was a main effect of task, *F*_(1, 15)_ = 5.39, *p* < 0.001, with significantly more blocks in the non-product condition, and a main effect of size, *F*_(3, 45)_ = 83.83, *p* < 0.001.

### 3.2. Testing

#### 3.2.1. Testing trials (all)

**Figure 5:**
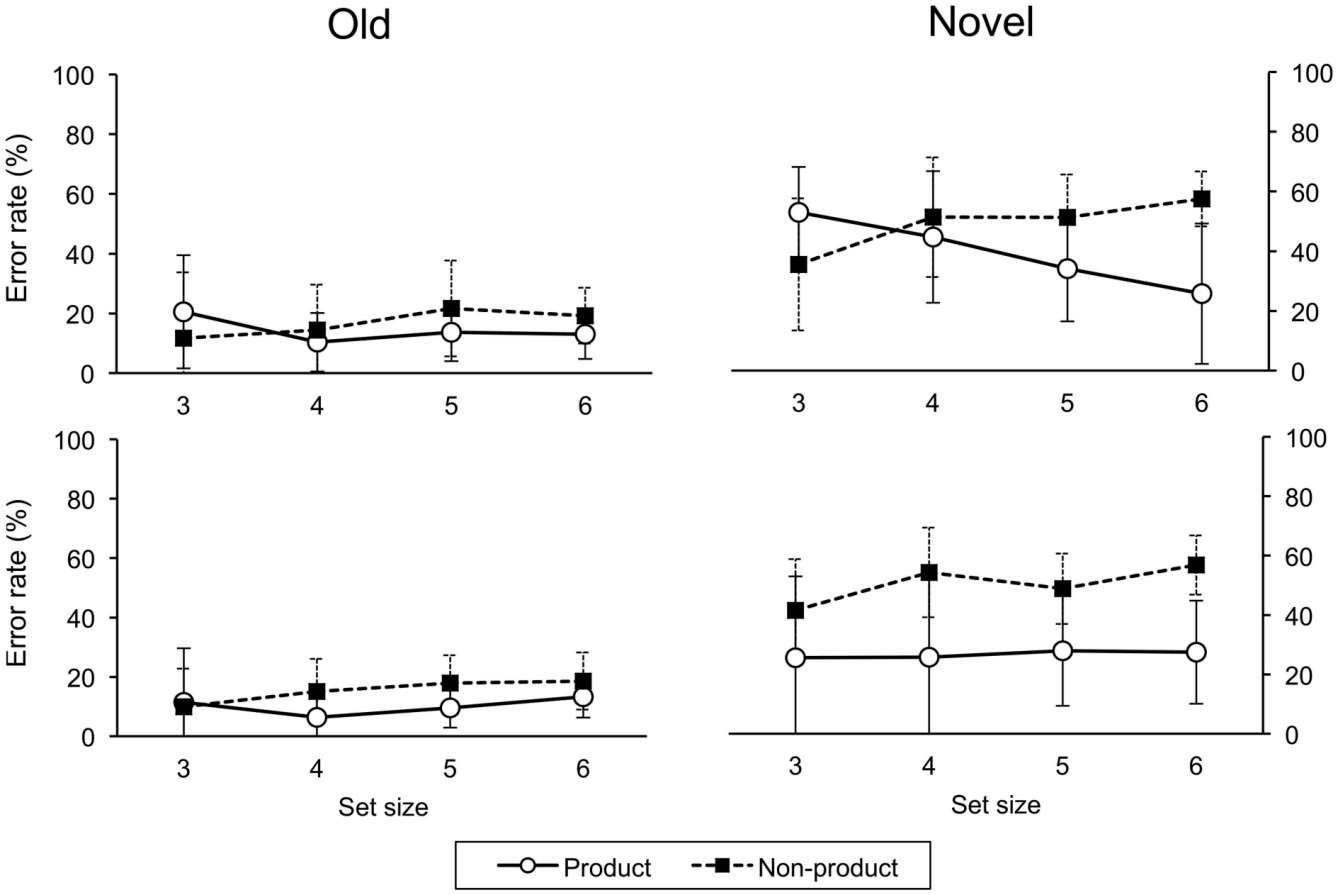
**Error rates on testing trials for ascend (upper panel) and descend (lower panel) groups**. Error bars indicate one standard deviation.

A four-factor (order, task, size, cue) ANOVA on error rate revealed main effects for order, *F*_(1, 29)_ = 4.39, *p* < 0.05, and task, *F*_(1, 29)_ = 28.36, *p* < 0.001. There were more errors in the ascend than descend order group; there were more errors in the non-product than product task. There were significant two-way interactions of order-task, *F*_(1, 29)_ = 4.95, *p* < 0.05, task-cue, *F*_(1, 29)_ = 13.06, *p* < 0.01, and task-size, *F*_(3, 87)_ = 12.70, *p* < 0.001. There was also a significant three-way interaction of order-task-size, *F*_(3, 87)_ = 3.88, *p* < 0.05. One-sample *t*-tests against chance level revealed significantly below chance level error rates:

– on old trials in product and non-product tasks at all set sizes for ascend and descend groups,
– on novel trials in the product task at all set sizes for the descend group, and
– on novel trials in the product task at set size six for the ascend group

(*p* < 0.05, Holm-Bonferroni correction for multiple comparisons). Error rates are shown in Figure 5.

#### 3.2.2. Novel and old trials

For novel trials, a three-factor (order, task, size) ANOVA on error rate revealed a main effect for task, *F*_(1, 29)_ = 28.57, *p* < 0.001. Errors were greater in the non-product than product condition. There were significant two-way interactions of order-task, *F*_(1, 29)_ = 5.21, *p* < 0.05, and task-size, *F*_(3, 87)_ = 10.22, *p* < 0.001. There was a significant three-way interaction, *F*_(3, 87)_ = 4.12, *p* < 0.05. For old trials, a three-factor (order, task, size) ANOVA on error rate revealed a significant two-way interaction of task-size, *F*_(3, 87)_ = 3.09, *p* < 0.05.

#### 3.2.3. Product and non-product tasks

For the product task on novel trials, a two-way (order, size) ANOVA revealed a main effects for order, *F*_(1, 29)_ = 4.36, *p* < 0.05, and size, *F*_(3, 87)_ = 3.38, *p* < 0.05, however, the effect of size was not significant after a correction for multiple comparison (Holm-Bonferroni method). There were more errors in the ascend than descend group. There was a significant interaction, *F*_(3, 87)_ = 4.74, *p* < 0.01. For the non-product task on novel trials, there was a main effect of size *F*_(3, 87)_ = 7.66, *p* < 0.001.

#### 3.2.4. Awareness (training)

With regard to the product task, 21 participants reported awareness of the product structure. The other 10 participants reported no awareness. The numbers of aware and unaware participants were (respectively) 11 and 4 for the ascend group, and 10 and 6 for the descend group. The mean number of blocks to criterion for the product task was larger in the unaware than aware ascend and descend groups. However, these differences were not significant.

#### 3.2.5. Awareness (testing)

**Figure 6:**
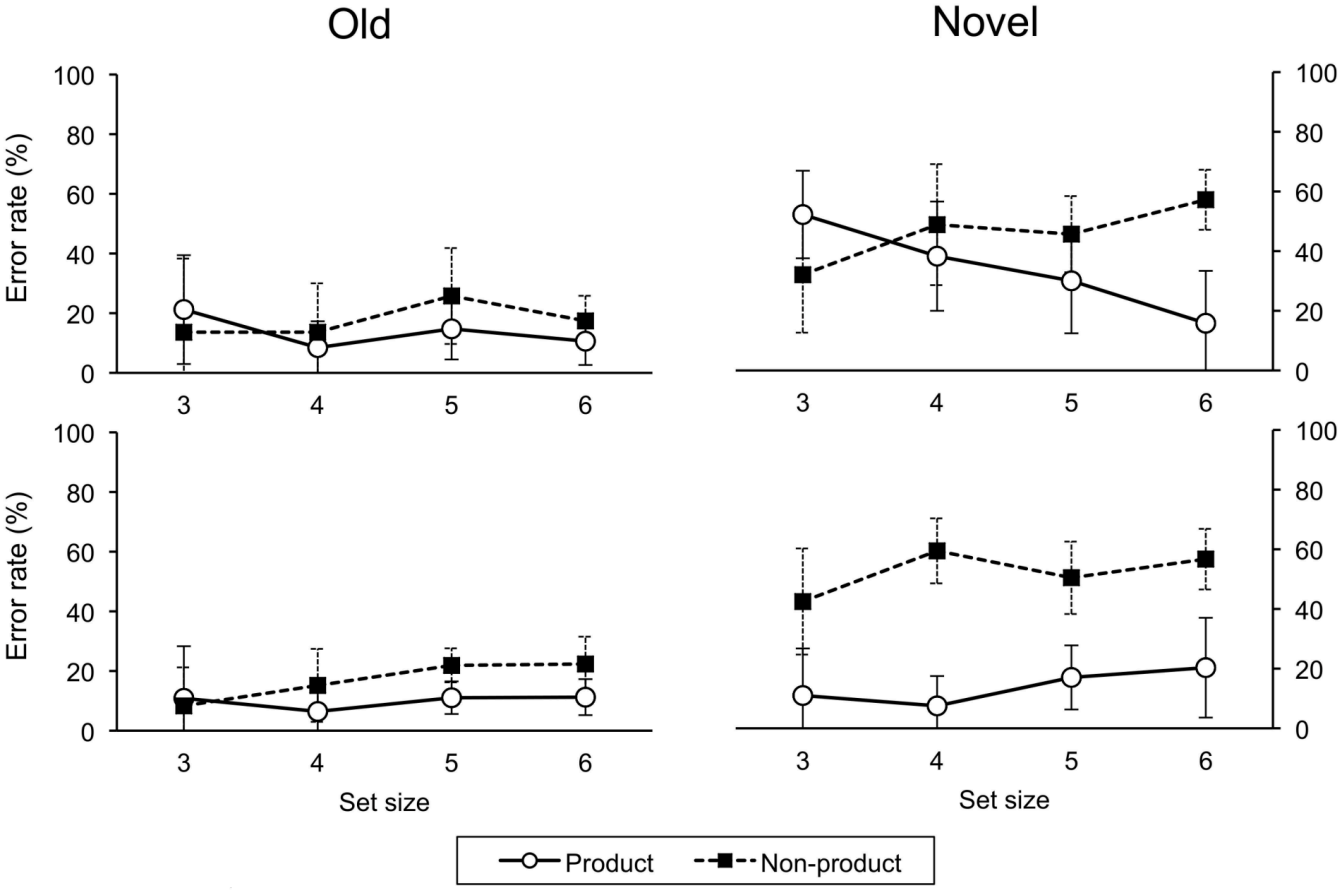
**Error rates on testing trials for ascend (upper panel) and descend (lower panel) aware groups**. Error bars indicate one standard deviation.

**Figure 7:**
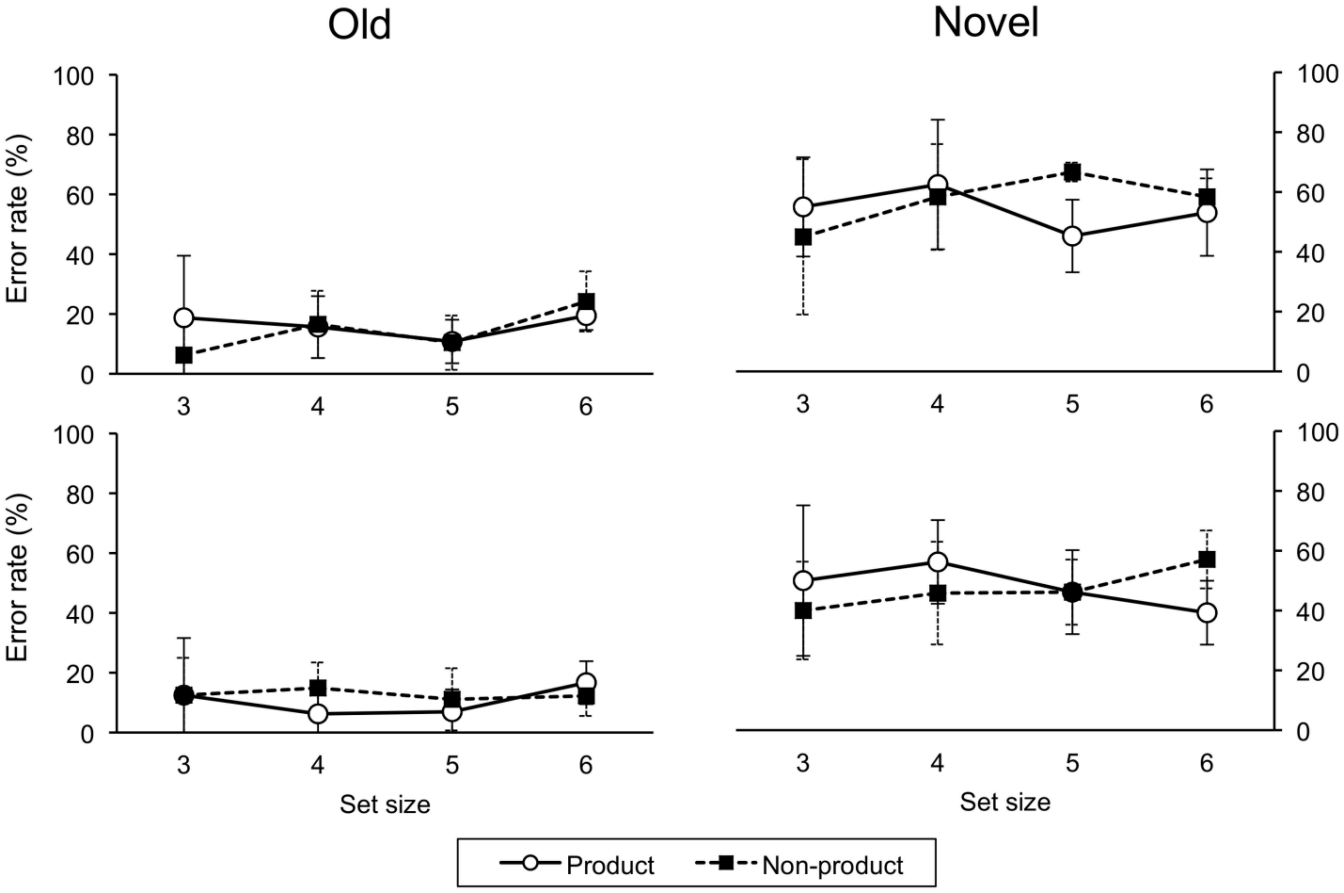
**Error rates on testing trials for ascend (upper panel) and descend (lower panel) unaware groups**. Error bars indicate one standard deviation.

For error rates on novel trials, a four-factor (Order, Awareness, Task, Size) ANOVA revealed a main effect of awareness, *F*_(1, 26)_ = 25.21, *p* < 0.001, the unaware group had more errors than the aware group (Order and task effects were also significant). Accordingly, we analyzed the aware and unaware data separately. For the aware group, a three-factor (Order, Task, Size) ANOVA revealed a main effect of task, *F*_(1, 18)_ = 129.70, *p* < 0.001, with more errors on the non-product than product task. There were significant two-way interactions of order-task, *F*_(1, 18)_ = 36.81, *p* < 0.001, and task-size, *F*_(3, 54)_ = 10.20, *p* < 0.001, and a significant three-way interaction, *F*_(3, 54)_ = 6.88, *p* = 0.001 (Figure 6). For the unaware group, there were no significant main effects or interactions (Figure 7).

**Figure 8:**
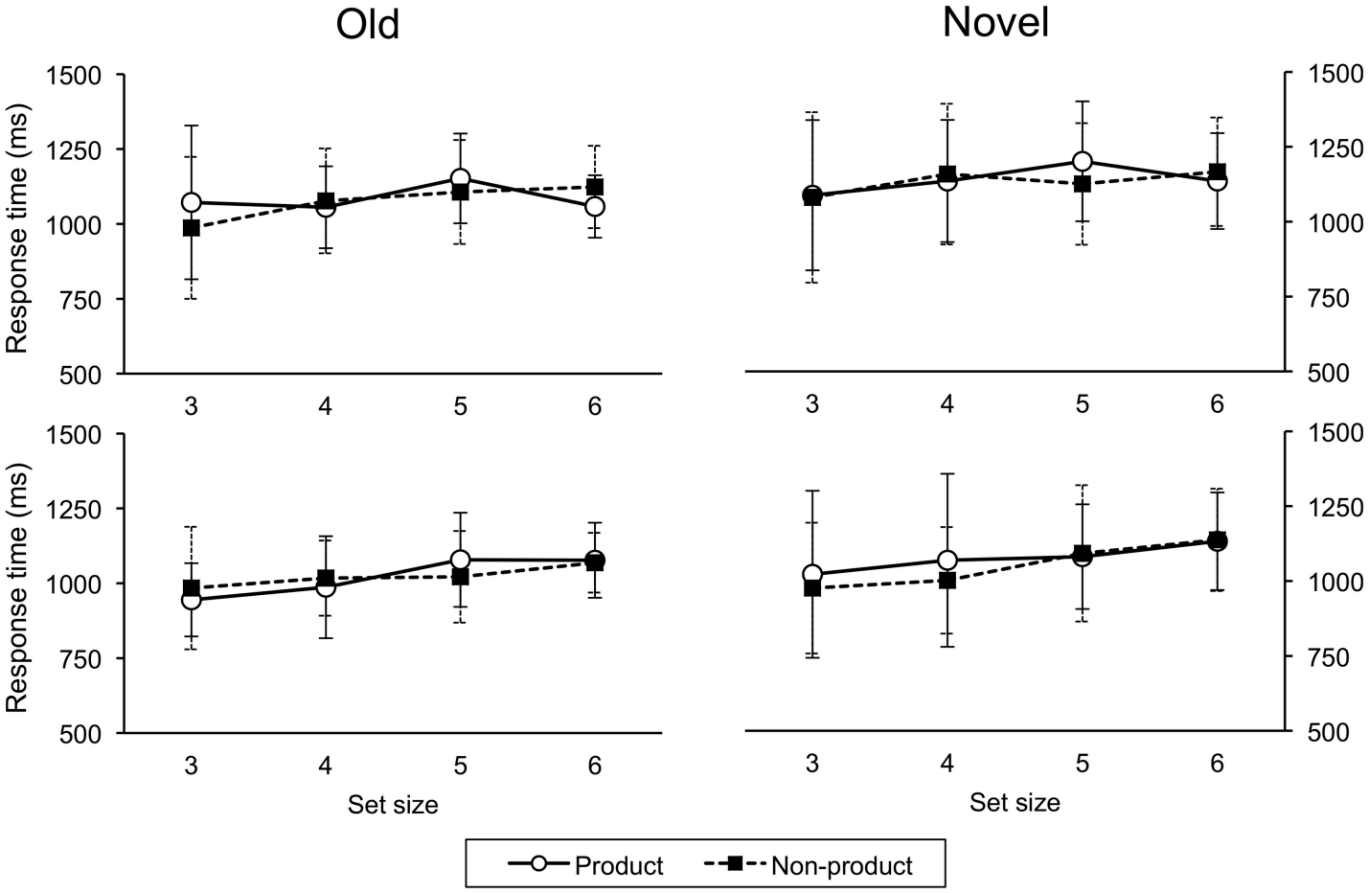
**Response times on testing trials for ascend (upper panel) and descend (lower panel) groups**. Error bars indicate one standard deviation.

## 4. Discussion

The prediction that universal construction, hence systematicity, depends on a cost-benefit tradeoff was supported by the significant three-way interaction of order-task-size on error rates for novel test trials. Recall that low error rates on novel test trials indicate behavioral generalization, which is possible in the product, but not the non-product task, i.e., tasks of the form *f*×*g*, but not 〈*p, q*〉. The significantly below chance level error rates on old test trials indicate that both ascend and descend groups of participants were able to learn both product and non-product tasks at all set sizes. For novel trials in the non-product task, performance was not significantly below chance, naturally, since no prediction on novel trials was logically possible. Importantly, generalization on the product task was observed across all set sizes for the descend group only. In the ascend group, performance was significantly lower than chance for set size six only, suggesting that participants learned cue-target responses as direct associations, i.e., without reference to the product structure. Mean error rates decreased (generalization increased) with increased set size, indicating that participants gradually performed the task with reference to the product structure.

The difference between ascend and descend groups cannot be explained by task difficulty, as measured by number of cue-target mappings, because performance for the ascend group was significantly lower than chance at the larger set size on the product task, whereas number of mappings implies the opposite effect, i.e., more errors with larger sets.

The difference between ascend and descend groups can be explained by a perceived (empirical) cost-benefit tradeoff, as mentioned in the Introduction (see also Phillips, 2013). The descend group is initially faced with learning a large number (18) of cue-target mappings. The effort needed to learn these mappings is lessened by exploiting the underlying product structure. In this case, one only need learn the six character-to-color and six character-to-shape mappings. Once this shortcut is observed, it can be applied to the other product tasks, hence generalization performance did not change with size for the descend group. By contrast, for set size three, only four cue-target mappings had to be learned, which is less than the three character-to-color plus three character-to-shape mappings. So, for the ascend group, who have yet to be exposed to the testing component of the task, there is no clear benefit in learning the training mappings as a product construction. Participants in the descend group, on the other hand, have already been exposed to the testing component for the product task at the other set sizes. Thus, although there is also no gain on training for the descend group, there is an expected performance gain in the testing component of the product task that is expected to follow.

Training performance, in terms of number of blocks to criterion, revealed that significantly fewer blocks were required in the product case in both ascend and descend groups, which further supports the learning of product constructions. Moreover, the order-size interaction is also consistent with the idea that the ascend group took advantage of product structure realized at smaller set sizes to make learning more effective at the size six. However, the interaction with task was not significant. One possible reason is the high variance: some participants required many blocks to reach criterion. Why fewer training blocks were needed at size five than four is unclear.

The self-report analysis also supported the claim that systematicity depends on an empirical cost-benefit tradeoff. The interaction of order-task-size was evident in the aware group, but not the unaware group. For the unaware group, generalization was basically flat across sizes and did not differ across task. This result further supports the importance of perceived cost-benefit tradeoff. However, the data do not allow us to determine the cause of awareness.

### 4.1. Categorical perspective

From a category theory perspective, the absence of a systematicity property is a consequence of failure to induce the associated universal construction. To understand failure to induce a universal constructions we need to understand the goals of the system in relation to task demands. The primary goal for all participants is to provide the correct response to a given cue. For the ascend group, this goal can be fulfilled in small set size conditions by simply learning the direct cue-target mappings. However, for larger set size conditions the resources (time, effort) needed to learn more mappings becomes burdensome, and so an alternative is sought. At some point, presumably, participants observe that the characters are mapped independently of each other which affords more effective learning via the product construction.

An important question, then, is what engenders the observation that the cue-target maps conform to a product. Note that even for large set sizes participants are still able to learn all cue-target mappings directly, as evidenced by their performance in the non-product condition across all set sizes. Thus, task demands alone cannot explain the shift toward products. Furthermore, task demands alone cannot explain failure of systematicity for the ascend group in small sized conditions, because the descend group did not employ a direct map approach in this condition, as evidenced by their significantly below chance level performance.

One possibility is that participants are sensitive to the co-occurrences between stimulus dimensions (i.e., left/right character, color, and shape), not just the co-occurrences of specific stimuli. An explanation that relies only on the co-occurrence of specific stimuli is perplexing because it suggests that products over small sets should more likely engender induction. For instance, to see that characters are mapped independently one needs trials, in close temporal proximity to minimize forgetting, where (say) a left character is paired with different right characters yielding the same target color. From such trials one can deduce that the mapping of the left character to a color does not depend on the right character. However, for larger sets there are more character combinations, hence the likelihood of the same character appearing in the same position on consecutive trials is less than that for small sets. That is simply because randomly selected consecutive pairs are less likely to share a common constituent in the larger set conditions. At the level of stimulus dimensions, however, large set sizes provide more examples of the relationship between dimensions that is independent of specific stimuli: e.g., that the color feature dimension is associated with the left character dimension, or that the shape feature dimension is associated with the right character dimension. Thus, the link between constituent sets, which is essentially the role or position that each constituent plays within the complex entity, is more informative in the large size conditions. Hence, induction of products on the basis of set-level co-occurrences is more likely to occur in the large size conditions, which is consistent with the data.

A response via a universal construction is composed of two constituent capacities (i.e., two maps) compared to a direct response which involves just a single map. This difference suggests that response times should be shorter in situations that do not involve computing a universal construction. However, participants must first correctly recognize the stimulus pair. This recognition process may take more time in the non-product case (*n*^2^) than the product case (2*n*) simply because the greater number of stimuli likely increases their similarity which makes them more difficult to discriminate. An analysis of response time data (see Appendix) revealed a significant effect for size (and cue, but not order or task) that is consistent with this possibility: the shorter response times for smaller set sizes suggest that any temporal advantage afforded by directly mapping stimuli to responses is offset by the additional time needed to determine the stimulus being presented.

### 4.2. A new challenge

From a computational learning perspective, where learning is treated as a search over a parameter space for a function that “best” fits the data, one can improve generalization by constraining search. A basic tradeoff is that some parameters afford a better fit to the given training data at the possible expense of poorer generalization on the new instances. In a probabilistic setting, this tradeoff is called the *bias/variance dilemma* (Geman et al., 1992). The available methods are too numerous to mention, but the basic idea is to guide search to regions more likely to contain “good” parameters. Many early connectionist attempts to address the systematicity challenge were essentially along this line, i.e., careful crafting of neural network models to demonstrate systematicity as a particular capacity for generalization (see, e.g., Hadley, 1994; Niklasson and van Gelder, 1994; Hadley and Hayward, 1997; Boden and Niklasson, 2000). A capacity to generalize is a well-known property of many kinds of neural networks. However, there are many ways to configure a network to learn, and not all of these ways afford the requisite level of generalization (see, e.g., Marcus, 1998; Phillips, 1998). Thus, a challenge for the learning approach is to explain why a network is configured in just the right way to afford the desired generalization property, which echoes the original systematicity problem. One can see this problem as a kind of *second-order systematicity* challenge (Aizawa, 2003): explain why an ability to *learn* one cognitive capacity implies an ability to learn another structurally related cognitive capacity. In the current context, that systematicity challenge is to explain why having the ability to learn one product map implies having the ability to learn another product map.

The results presented here, however, raise a further challenge because for the ascend group on the product series the capacity to learn the product map at set size six did not imply the capacity to learn the product map at set size three. The further challenge is to explain why under some conditions the cognitive system possesses a systematicity property and under other (closely related) conditions it does not. From a category theory perspective, one possible way forward is a category theory treatment of (co)recursion (see, e.g., Arbib and Manes, 1975; Bird and de Moor, 1997), in which case a system can be treated as a generalized state machine (Rutten, 2000). Suppose learning is considered as a recursive process: a process that maps (generalized) cognitive states that include the currently available cognitive capacities to cognitive states that include possibly newly acquired cognitive capacities. A category theory treatment of recursion, via certain kinds of universal constructions, called *initial algebras* and *final coalgebras*, may provide a basis for explaining the systematicity of learning (Phillips and Wilson, 2016b). For instance, every universal construction is the “optimal” construction, in a particular category theoretic sense, which can be learned from a general optimization procedure (Phillips and Wilson, 2016a). Yet, the results presented here suggest that any approach to the systematicity of learning will have to take into account the goals of the cognitive system, the costs and benefits (in terms of cognitive resources) of learning a cognitive capacity with vs. without representing the underlying universal construction, and prior experience to meet this new challenge.

Learning alternative strategies in response to changes in context also raises broader issues for models of cognition, such as how the acquisition of new strategies interacts with previously acquired strategies. Marchiori and Warglien (2011) report that in a game scenario participants can learn and choose between different strategies in response to changes in their opponent's strategy. In contrast to humans, neural network models with neural activity based on a *sigmoidal* function tend to “average” responses. However, this shortcoming can be addressed with neural activity based on a *softmax* function (Marchiori and Warglien, 2011), which is a generalization that affords the learning of multimodal (as opposed to unimodal) distributions. For example, this approach has been used to model a mixture of experts (Jacobs et al., 1991). Such models may also help address the new systematicity challenge raised by the current study. For the current study, however, our experimental design and data do not allow us to investigate such interactions. Although response times were shorter for old than novel cues during the testing phase of the product condition (i.e., after learning the universal construction), see Appendix, suggesting that participants switched between product and non-product strategies based on cue, this difference can also be explained as a practice effect. Further work is needed to determine how the acquisition of strategies based on universal constructions interact with other strategies.

## Author contributions

All authors listed, have made substantial, direct, and intellectual contribution to the work, and approved it for publication. SP conceived the study; SP, YT, and FS designed the experiment; FS implemented the experiment and collected the data; SP, YT, and FS analyzed the data; and SP, YT, and FS wrote the manuscript.

## Funding

This work was supported by a Japanese Society for the Promotion of Science Grant-in-aid (26280051).

### Conflict of interest statement

The authors declare that the research was conducted in the absence of any commercial or financial relationships that could be construed as a potential conflict of interest.

## Appendix

*Testing trials (response times)*: A four-factor (order, task, size, cue) ANOVA on response times (for error-free testing trials) revealed main effects for size, *F*_(3, 87)_ = 6.31, *p* < 0.005, and cue, *F*_(1, 29)_ = 8.56, *p* < 0.01. Response times were shorter for size 3 than sizes 5 and 6, and shorter for old than novel trials. There was no effect of order, *F*_(1, 29)_ = 1.90, *p* = 0.18, or task, *F*_(1, 29)_ = 0.23, *p* = 0.64. There were no significant interactions. Response times are shown in Figure 8.
